# Polyol Synthesis: A Versatile Wet-Chemistry Route for the Design and Production of Functional Inorganic Nanoparticles

**DOI:** 10.3390/nano10061217

**Published:** 2020-06-22

**Authors:** Souad Ammar, Fernand Fiévet

**Affiliations:** Université de Paris, CNRS UMR-7086, ITODYS, 15 rue Jean Antoine de Baïf, 75205 Paris, France

The term “polyol process” was first used in the late eighties by Fiévet, Lagier, and Figlarz [1,2,3] as a liquid-phase synthesis route to obtain finely divided metals from their oxides, hydroxides, or salts in polyalcohols. The basic concept was to prepare metal powders using a liquid organic compound acting both as a solvent of the solid precursor and as a reducing agent. Polyalcohols such as α-diols and ether glycols, resulting from their condensation, appeared very convenient for this purpose. In this initial context, polyols were able to reduce to zero-valent state ions of noble metals, copper, and also more electropositive metals such as cobalt and nickel. From these first studies, it was foreseen in the early nineties that the polyol process could be a versatile and promising method for the synthesis of metal and alloy powders made up of non-agglomerated particles with a well-defined shape, narrow size distribution, and controlled size in the micrometer or submicrometer [4,5] range and, in some cases, as colloidal dispersions [6,7]. It was also clearly evidenced in this pioneering work that these interesting morphological characteristics were the result of the kinetic control of a multi-step process: the dissolution of a solid precursor, nucleation, and particle growth. It was shown that in some cases, this control can be more easily achieved if homogeneous (spontaneous) nucleation is replaced by heterogeneous nucleation by seeding the reactive medium with foreign nuclei [1,2,3].

A first example of shape control was given in the polyol-mediated synthesis of silver particles from silver nitrate [6]. Whereas quasi-spherical and monodisperse silver particles were produced through spontaneous nucleation, provided particle sintering was prevented during the growth step by adding polyvinylpyrrolidone (PVP) as a protective agent, the heterogeneous nucleation of metallic silver with a critical concentration of in situ-formed platinum nuclei produced mono-sized particles with a rod-like shape. By adsorbing on a specific plane of the foreign nuclei’s surface, the protective agent also acts as a crystal-habit modifier and induces the anisotropic growth of the particles. All these features and others were then extensively studied by Xia et al., succeeding in reaching a total control of silver nucleation and crystal growth in polyol and in producing on demand particles of different sizes and shapes [7,8,9].

The next development of the polyol process began with the synthesis of monodisperse submicrometer spherical zinc oxide particles from zinc acetate dihydrate in diethyleneglycol [10]. To obtain oxides, water is required and can be provided either by using hydrates as starting materials or by adding definite amounts of water. The reaction is carried out at a temperature close to the boiling point of the mixture in order to favor forced hydrolysis, followed by inorganic polymerization via olation and oxolation reactions. Then, Feldmann et al. evidenced that this polyol-mediated preparation method was able to provide nanoscale oxide particles with a mean size in the range 30–300 nm, with a low degree of agglomeration and a monodisperse size distribution; the resulting colloidal suspensions containing up to 20 wt.% solids were found to be stable [11]. Later, Feldmann et al. extended the polyol route to the synthesis of many oxides to obtain nanoscale functional materials, such as luminescent materials (phosphor host lattices: e.g., Y_2_O_3_), pigments (CoAl_2_O_4_, Cr_2_O_3_, ZnCo_2_O_4_, Ti_0.85_Ni_0.05_Nb_0.10_O_2_), transparent conductive oxides (ZnO:In^3+^), and catalytically active oxides (CeO_2_, Mn_3_O_4_, V_2_O_5_) [12]. At the same time, Ammar et al. carried out the first synthesis of a spinel-like ferrite (CoFe_2_O_4_) by forced hydrolysis in 1,2-propanediol [13]. They obtained monodisperse nanoparticles (NPs) showing a surprisingly good crystallinity and a high saturation magnetization. Among various methods of fabrication of ferrite NPs, the forced hydrolysis of metal salts in polyol appears therefore as a new and attractive soft chemistry route. On the one hand, like the microemulsion method, this allows one to control the nucleation and growth steps of the particles. The adsorbed organic species protect the primary particles against coalescence and/or aggregation. On the other hand, this crystal growth occurs under thermal conditions close to hydrothermal ones (in the range 150–225 °C under reflux). As a consequence, a good crystallinity is achieved, which in turns results in an improved magnetic order with a high saturation magnetization. This work paved the way to the synthesis by the polyol process of a large variety of nanoscaled ferrites and to the study of their structural and magnetic properties [14,15,16,17,18,19,20,21] and, in some cases, the study of the oxidation state of the involved cations and their catalytic properties [22].

Simultaneously, by increasing the amount of added water in the polyol medium, Jouini et al. obtained by hydrolysis and condensation reactions layered hydroxide acetate salts M(OH)_1−*x*_(CH_3_COO)_x_·*n*H_2_O (M = Co, Ni, Co-Ni and Zn) [23]. The compounds obtained present the typical features of the brucite-like structure, with turbostratic disorder and an interlayer spacing ranging around 0.1 nm. Then, by exchanging acetate ions for halides [24] and *n*-alkylsulfonates C*_n_*H_2*n*+1_SO_3_^−^ with *n* ~ 10–18 [25], they formed a series of derivatives with various interlayer spacing, ranging between 0.7 to 3.2 nm, forming a novel series of magnetic layered materials for which the model of ferromagnetic layers interacting through dipolar coupling can be applied [24,25].

Double hydroxide layers, typically those with NiAl and CoAl compositions, were also prepared in polyols, with the advantage, over the standard co-precipitation method, of phase purity and a controlled morphology [26].

At this stage, the polyol process appeared to be a new route for the preparation of powdery metal, oxide, and hydroxide materials. It was shown through the cobalt example that the use of acetate precursors, contrary to chlorides or sulphates, leads to the precipitation of a solid (metal, oxide, hydroxide) whose nature depends on a main factor: the hydrolysis ratio *h*, defined by the water to metal molar ratio [27].

As in the sol-gel method, acetate leads to the formation of intermediate alkoxyacetate complexes. The absence of water favors metal formation, while its presence favors hydroxyacetate and oxide at higher temperatures. Compared to the classical sol-gel route, the polyol-mediated synthesis offers several advantages: the use of common ionic salts instead of alkoxides as precursors; a wide operating temperature range making it possible to obtain oxides and metals with good crystallinity.

More recently, more complex oxides such as molybdates and tungstates [28,29] have been successfully obtained in polyols without further thermal annealing.

Extrapolating the hydrolysis reaction to the larger nucleophilic substitution concept, other than hydroxides and oxides, inorganic compounds were produced by the polyol process. Introducing in the reaction medium the desired nucleophilic agents—S^2−^, PO_4_^3−^ or F^−^, for instance—allowed, for instance, the preparation of size-controlled chalcogenide [30,31,32,33,34], phosphate [35,36,37], and fluoride [38,39] particles. Nucleophilic substitution and condensation reactions allowed also the precipitation of well-shaped and size-controlled metal glycolate crystals, which can themselves serve as experimental models for the study of some theoretical electromagnetic features [40,41].

This list has been incremented by the pioneer work of Feldman et al., who first prepared Carbon dots, a new generation of solid chromophores, by the thermal decomposition of polyols [42].

Clearly, the polyol process is emerging now as a powerful and scalable wet chemistry route for the production of a large variety of chemically, structurally, and morphologically controlled inorganic nanoparticles. Through a simple optimization of the operating synthesis conditions, it allows the design of well-shaped granular and nanostructured metals, oxides, chalcogenides, halides, alkoxides, hydroxides, and others, with a great applicative interest for various technological fields, including renewable energy, human health, environment, microelectronic, photonic, etc.

These particles have been also used as seeds, when dispersed in a metallic salt polyol solution, to grow original hetero-nanostructures [43,44,45,46] with synergetic and improved functional properties, thus offering a larger panel of original materials for the listed above applications.

They have been used also as starting matter for the fabrication of granular nanomaterials that are new by composition, structure, or microstructure, thanks to a judicious subsequent treatment, like oxidative [47,48] (or reductive [49,50]) annealing, cold plasma implantation [51] and reactive sintering [52,53].

Selected recent results dealing with all these aspects, at least some of them, are highlighted in this series. Typically, Lehmacher et al. [54] present a polyol-mediated synthesis of nitrogen-containing C-dots with 1,2,4,5-tetracyanobenzene as a starting material. This one-pot liquid-phase synthesis provides, with a good reliability, particles with interesting features—such as a narrow size distribution (2–4 nm), high crystallinity, high dispersibility in water, and deep-red fluorescence—which may be used as emitting biomarkers for histology. Another example of a polyol-mediated synthesis of nanoparticles for bio-imaging applications is that illustrated by the work of Becerro et al. [55]. Indeed, they succeeded in producing, in polyol, nanometer-sized Eu^3+^:(H_3_O)Lu_3_F_10_ single crystals. By varying the Eu content, they obtained particles with an optimized luminescence response and X-ray attenuation capacity for their use as bimodal probes for optical imaging and X-ray-computed tomography. To date, such a synthesis has never been reported. The Eu^3+^:(H_3_O)Lu_3_F_10_ compound had only been fabricated in the form of polycrystalline powders [56,57].

Similarly, pure ovoid-like shaped bismuth ferrite nanoparticles were successfully synthesized for the first time in polyol by Coste et al. [58]. The synthesis parameters were controlled to obtain an average particle size of 40 nm, which is under the periodicity of the modulated cycloidal arrangement of iron spins. Due to their small size, the as-obtained particles exhibit a transformation of the overall magnetic order and an exalted spontaneous magnetization compared to micrometer-sized particles. The resulting improvement of the magnetoelectric coupling makes these nanostructured bismuth ferrite powders low-cost potential candidates for spintronic applications.

Enhanced photochromic properties have been obtained as well with polyol-made WO_3−*x*_ nanoparticles. By varying the polyol synthesis parameters and the post annealing conditions, Gaudon [59] et al. prepared WO_3−*x*_ nanoparticles with a very broad range of coloration from black to yellow (through blue-green colorations) and evidenced in these particles photochromic behavior highly dependent on their composition and color. Rapid and important changes in coloration under UV irradiation were especially observed on the blue compound, this photochromism being reversible in a few hours. The developed polyol chemical route clearly offers new perspectives for the use of tungsten oxides as smart photochromic compounds.

Jouini et al. [60] exemplify the ability of polyol-mediated synthesis to control the shape and size of Cobalt nanorods. By applying an external magnetic field during the synthesis, they produced anisotropic in shape metal particles of different lengths and aspect ratios. They also succeeded by this way in avoiding conical heads at the extremities of these 1D nanomagnets, leading to assemblies with greatly improved magnetic properties. Fujieda et al. [49] proposed another strategy in order to prepare 1D nanomagnets. They prepared iron glycolate nanowires in polyol and they annealed them subsequently under moderate reductive conditions. They showed that it is possible to produce in this way ferrimagnetic magnetite and/or ferromagnetic iron while maintaining the one-dimensional shape of their starting material.

Another original result concerns the direct synthesis of “iron particles” in polyol. Usually, this method allows the size control of these particles through the use of hexachloroplatinic acid as a nucleating agent and the variation in its content in the reaction medium [61]. Although the concentration of Pt ions was low, it led to appreciable cost effects for any technological application. Thus, other routes to reduce the particle sizes of polyol-synthesized iron should be considered. Such an approach is proposed by Leybo et al. [62] by adding ascorbic acid as a more powerful reducing agent to the NaOH-polyol reacting medium. Iron powders, Fe/graphene oxide, and Fe/boron nitride composites were so-synthesized, and the effect of the NaOH/Fe and ascorbic acid/Fe ratios on the characteristics of the synthesized products were evaluated. The addition of NaOH results in the formation of a passivation layer mainly composed of sodium carbonates, protecting the iron particles from irreversible oxidation. The addition of ascorbic acid leads to the 10-fold decrease in the size. Tested in wastewater treatment, these iron particles exhibit a 2-fold increase in the efficiency of lead removal from wastewater.

The polyol-mediated synthesis of other composite powders has been also reported. Mnasri et al. [63] succeeded to prepare in polyol multifunctional granular hetero-nanostructures based on lanthanide doped fluoride single crystals coated by superparamagnetic iron oxide nanosatellites. Dispersed as a colloid in water, the resulting composite particles evidence improved optical and magnetic properties, as well as reasonable biocompatibility and low toxicity to be used as bimodal agents for biomedical magnetic resonance and optical imaging.

Nguyen et al. [64] propose another polyol-mediated preparation of biocompatible multifunctional hetero-nanostructures. They succeeded in building composite nanoparticles based on a polycrystalline magnetite-like core surrounded by a star-shaped gold nanosatellite, exhibiting interesting magnetic and plasmonic properties for magnetically assisted Surface-Enhanced Raman Scattering (SERS) detection.

Finally, Haj-Khlifa et al. [51] use polyol-made nano-metals as starting materials for the production of crystallized nano-intermetallics by hydrogen cold plasma implantation. They evidence that the nanometric size of nickel particles, even compacted as a thick pellet (millimeter in thickness) allows the diffusion of atomic hydrogen and its interaction with nickel atoms to form, within optimized processing conditions, Ni_2_H nano-intermetallics crystallizing in a hexagonal structure, paving the way for an alternative route for the preparation of efficient substrates for solid hydrogen storage application.

Considering the high applicative potential of all these polyol-made nanoparticles and others, and to be as exhaustive as possible in our approach, the ecotoxicological impact of such materials is addressed. Brayner et al. [65] investigate, for instance, the fate of polyol-made ZnO and CdS nanoparticles (NPs) in Seine river water (Paris, France) in the presence of *Chlorella vulgaris* microalgae. They first observe the internalization of these particles after 48 h of contact with *Chlorella vulgaris* at doses of 10^−3^ M in water. They also demonstrate by growth rate tests that the toxicity of these particles is higher than that of free Zn^2+^ and Cd^2+^ ions. These results have to be considered very seriously before any release of these particles in the environment and make us, as physicochemists of nanomaterials, more than any other scientist, particularly concerned with the subject.

So, to conclude, this review clearly illustrates that the polyol process offers the possibility to obtain a wide variety of granular homo- and hetero-nanostructures with optimized characteristics offering an unprecedented panel of properties for a direct and rapid technological applicative transfer. It also alerts us to their potential dangers if they are hazardously manipulated.
